# An allelic variant of the PmrB sensor kinase responsible for colistin resistance in an *Escherichia coli* strain of clinical origin

**DOI:** 10.1038/s41598-017-05167-6

**Published:** 2017-07-11

**Authors:** Antonio Cannatelli, Tommaso Giani, Noemi Aiezza, Vincenzo Di Pilato, Luigi Principe, Francesco Luzzaro, Cesira L. Galeotti, Gian Maria Rossolini

**Affiliations:** 1 University of Siena, Department of Medical Biotechnologies, Siena, 53100 Italy; 2 University of Florence, Department of Surgery and Translational Medicine, Florence, 50134 Italy; 3 Lecco A. Manzoni Hospital, Clinical Microbiology and Virology Unit, Lecco, 23900 Italy; 4 University of Florence, Department of Experimental and Clinical Medicine, Florence, 50134 Italy; 5 Florence Careggi University Hospital, Clinical Microbiology and Virology Unit, Florence, 50134 Italy

## Abstract

We investigated the colistin resistance mechanism in an *Escherichia coli* strain (LC711/14) isolated in Italy in 2014, from an urinary tract infection, which was previously shown to express a colistin resistance mechanism different from *mcr-1*. LC711/14 was found to carry a novel mutation in the *pmrB* gene, resulting in a leucine to proline amino acid substitution at position 10 of the PmrB sensor kinase component of the PmrAB signal transduction system. The role of this substitution in colistin resistance was documented by expression of the wild-type and mutated alleles in a *pmrB* deletion derivative of the *E. coli* reference strain MG1655, in which expression of the mutated allele conferred colistin resistance and upregulation of the endogenous *pmrHFIJKLM* lipid A modification system. Complementation of LC711/14 with the wild-type *pmrB* allele restored colistin susceptibility and decreased expression of *pmrHFIJKLM*, confirming the role of this PmrB mutation. Substitution of leucine at position 10 of PmrB with other amino acids (glycine and glutamine) resulted in loss of function, underscoring a key role of this residue which is located in the cytoplasmic secretion domain of the protein. This work demonstrated that mutation in this domain of the PmrB sensor kinase can be responsible for acquired colistin resistance in *E. coli* strains of clinical origin.

## Introduction

The emergence and dissemination of extensively drug-resistant (XDR) Gram-negatives (e. g. colistin-only susceptible *Pseudomonas aeruginosa*, carbapenem-resistant *Acinetobacter baumannii*, and carbapenem-resistant *Enterobacteriaceae*) in the clinical setting has led to the renewed use of colistin for treatment of infections caused by these pathogens^1^. Consequently, colistin resistance has been increasingly reported^2–4^. Thus far, the problem has mostly involved *Klebsiella pneumoniae* and *Acinetobacter*, while acquired colistin resistance has remained uncommon among clinical isolates of *Escherichia coli* ^5–8^. Conversely, colistin resistance has been reported more frequently among *E. coli* isolates of animal origin, likely due to the extensive use of colistin in veterinary medicine^9^.

In *E. coli*, resistance to colistin has mostly been associated with the acquisition of transferable *mcr*-type genes, which encode enzymes able to modify the lipopolysaccharide (LPS) colistin target by the addition of phosphoethanolamine^10–12^. Chromosomal mutations in the *pmrAB* genes, encoding a two-component signal transduction system which regulates the endogenous LPS modification systems, have also been described in colistin-resistant (Col-R) *E. coli* isolates from swine and poultry, but without a formal experimental confirmation of their role in conferring resistance to colistin^13^.

In this study we characterized a Col-R *E. coli* strain of clinical origin carrying a novel *pmrB* allelic variant encoding a PmrB protein with a leucine to proline amino acid substitution at position 10, and demonstrate that this mutation is associated with acquisition of colistin resistance in *E. coli*.

## Results

### Resistance and clonal profiles of *E. coli* strain LC711/14

*E. coli* LC711/14 was isolated in 2014 from the urine of an outpatient suffering from an urinary tract infection. Routine susceptibility testing, using the Vitek2 automated system, revealed that the strain was susceptible to all tested antibiotics (amoxicillin-clavulanic acid, cefotaxime, ceftazidime, cefepime, piperacillin-tazobactam, ertapenem, imipenem, meropenem, gentamicin, ciprofloxacin, trimethoprim-sulfamethoxazole) except colistin. Resistance to colistin was confirmed by reference broth microdilution, which yielded an MIC of 4 mg/L (Table 1). The patient had not been previously treated with colistin.

**Table 1:** Colistin MICs and expression levels of *pmrK* of different *E. coli* strains and transformants complemented with the pACYC-*pmrB*, pACYC-*pmrB*_t29c_, pACYC-pmrB_c28g/t29g_ and pACYC-*pmrB*_t29a_ plasmids.

Strain	Origin	MIC colistin (mg/L)	Chromosomal *pmrB* Locus	Episomal *pmrB*	*pmrK* expression^#^
MG1655	Keio collection	0.125	WT	none	1
MG1655_Δ*pmrB* (pACYC-*pmrB*)	This work	0.125	Deleted	*pmrB*_MG1655WT_	1.11 ± 0.2
MG1655_Δ*pmrB* (pACYC-*pmrB*_t29c_)	This work	4	Deleted	*pmrB*_MG1655t29c_ (Leu_10_Pro)	53.1 ± 26.7
MG1655_Δ*pmrB* (pACYC-*pmrB*_c28g/t29g_)	This work	0.125	Deleted	*pmrB*_MG1655c28g/t29g_ (Leu_10_Gly)	n.d.
MG1655_Δ*pmrB* (pACYC-*pmrB*_t29a_)	This work	0.125	Deleted	*pmrB*_MG1655t29a_ (Leu_10_Gln)	n.d.
LC711/14	Clinical strain	4	*pmrB*_LC711/14t29c_ (Leu_10_Pro)	none	83 ± 40.3
LC711/14 (pACYC-*pmrB*)	This work	1	*pmrB*_LC711/14t29c_ (Leu_10_Pro)	*pmrB*_MG1655WT_	4.1 ± 0.6
LC711/14 (pACYC184)	This work	4	*pmrB*_LC711/14t29c_ (Leu_10_Pro)	none	83 ± 15.4
LC711/14 (pACYC-*pmrB*_c28g/t29g_)	This work	0.125	*pmrB*_LC711/14t29c_ (Leu_10_Pro)	*pmrB*_MG1655c28g/t29g_ (Leu_10_Gly)	n.d.
LC711/14 (pACYC-*pmrB*_t29a_)	This work	0.125	*pmrB*_LC711/14t29c_ (Leu_10_Pro)	*pmrB*_MG1655t29a_ (Leu_10_Gln)	n.d.

^#^The fold differences obtained were normalized against the MG1655 values. n.d. not determined.

Multilocus sequence typing (MLST) analysis revealed that strain LC711/14 belonged to ST59, a lineage previously described to include strains with high virulence potential^14^.

### Colistin resistance mechanism in *E. coli* strain LC711/14: identification of a PmrB amino acid substitution involved in colistin resistance

LC711/14 was part of a collection of nine Col-R *E. coli* strains of clinical origin that were previously investigated to verify the presence of the *mcr-1* gene, and resulted the only strain negative for *mcr-1* ^15^.

Transcriptional analysis by quantitative real-time PCR (qRT-PCR) showed that *pmrK* expression in LC711/14 was increased compared to what observed in the reference wild-type *E. coli* strain MG1655, which is colistin-susceptible (Col-S) (Table 1). These results suggested that the Col-R phenotype of LC711/14 was consequent to upregulation of the *pmrHFIJKLM* operon, encoding the endogenous LPS modification system^16^.

**Figure 1:**
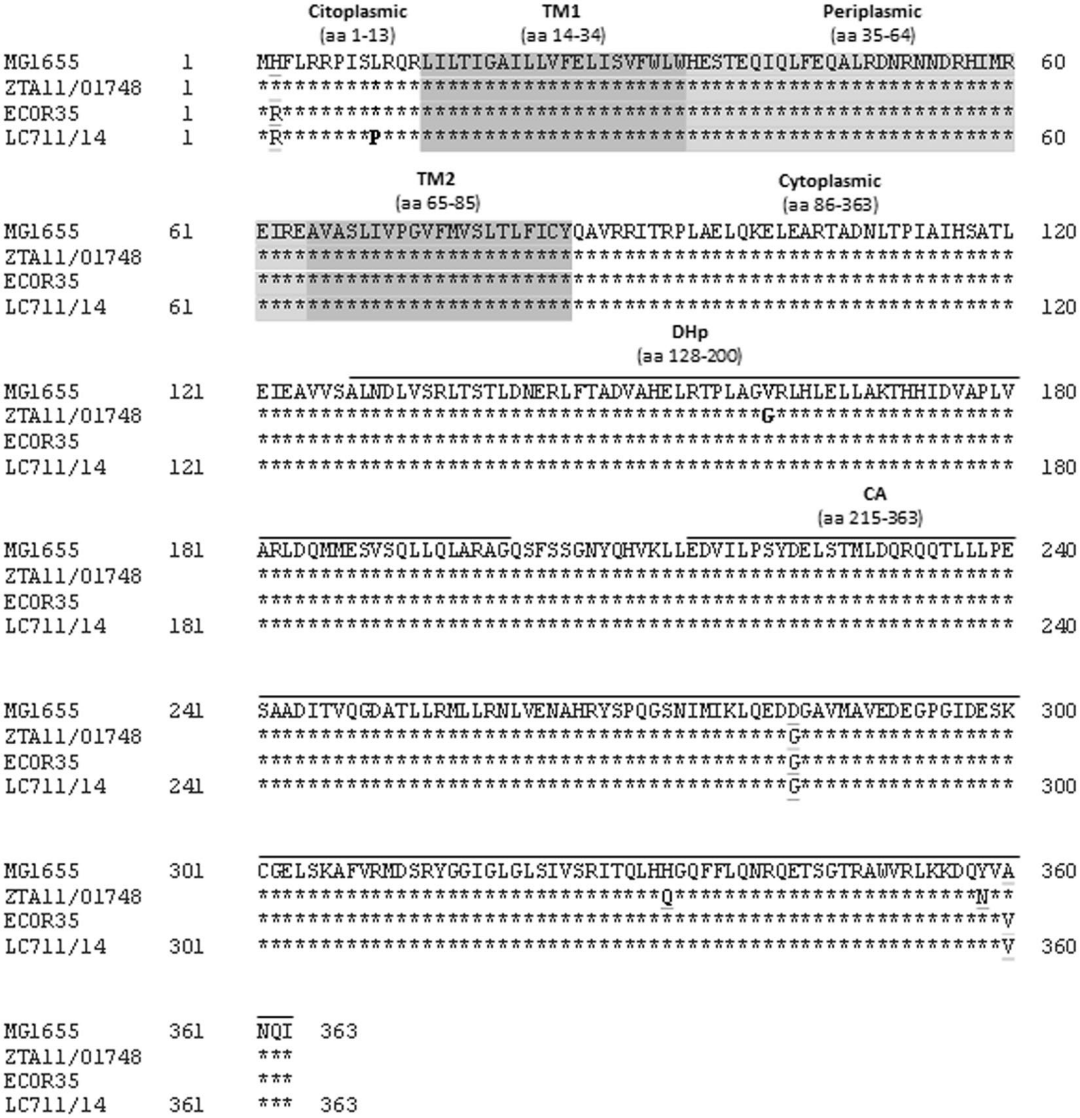
Protein sequence alignment of PmrB of *E. coli* MG1655 (accession no. NC_000913.3), ECOR35 (accession no. JN032071.1), LC711/14 (this work), and ZTA11/01748 (Col-R strain isolate from swine faeces, carrying a PmrB mutation putatively associated with colistin resistance^13^. Asterisks indicate conserved amino acid residues. Amino acids polymorphisms not associated with colistin resistance are underlined. The L10P substitution found in LC711/14 and demonstrated to be involved in colistin resistance in this work, and the V161G substitution described in a veterinary *E. coli* strain and putatively associated with colistin resistance are boldfaced. Provean (Protein Variation Effect Analyzer: http://provean.jcvi.org/seq_submit.php) analysis was carried out for the Leu_10_Pro (−2,67) and Val_161_Gly (−5.66) mutations, and indicated that both mutations had an impact on the PmrB topology. Structural and functional domains of the protein by Phobius prediction (http://phobius.sbc.su.se/) are also indicated: TM1 and TM2: transmembrane 1 and transmembrane 2 domains, are shaded in dark grey, while the periplasmic domain is shaded in light grey. The sub-domains (DHp: histidine phosphotransfer; CA: catalytic and ATP-binding) of the cytoplasmic portion of PmrB are overlined by black bars^32^. The number of residues of each domain are indicated in brackets.

Molecular characterization of the chromosomal *pmrAB* genes, encoding a two-component system known to be involved in the regulation of the *pmrHFIJKLM* operon^17^, revealed a t29c transition in *pmrB*, resulting in a non-synonymous leucine to proline amino acid substitution at position 10 (Leu_10_Pro) of the protein compared with other *E. coli* PmrB proteins present in the non-redundant nucleotide collection Blast database (including PmrB of MG1655) (Fig. 1). While some polymorphisms of the PmrB sequence exist among different *E. coli* strains, some of which have previously been putatively associated with a Col-R phenotype (e. g. Val_161_Gly)^13^ (Fig. 1), the Leu_10_Pro substitution was not previously reported. In fact, a leucine residue appeared to be strictly conserved at this position in 256 records of full-length *E. coli* PmrB present in the non-redundant protein sequences Blast database (accessed on April 18, 2017), and also in the PmrB sequences of the 8 *mcr-1*-positive Col-R strains of the collection from which LC711/14 was taken^15^. The closest PmrB variant present in the database was that of *E. coli* ECOR35 (Accession number AEL97557), which was identical to that of LC711/14 except for the Leu_10_Pro substitution (Fig. 1). Data regarding the colistin susceptibility of ECOR35 strain are not available.

To investigate the role of the PmrB Leu_10_Pro amino acid substitution in colistin resistance, the effect of this substitution was studied in the reference Col-S *E. coli* strain MG1655. The PmrB sequence of this strain shares 360/364 identical amino acid residues with that of LC711/14, including the Leu_10_ residue (Fig. 1). A *pmrB* deletion mutant of MG1655 (MG1655_Δ*pmrB*), complemented with the *pmrB* allele from MG1655 containing the t29c substitution (PmrB_MG1655_ Leu_10_Pro) exhibited a Col-R phenotype (MIC, 4 mg/L), while the same strain complemented with the wild-type *pmrB* allele (PmrB_MG1655WT_) remained colistin susceptible (MIC, 0.125 mg/L) (Table 1). Analysis of the expression of *pmrK* revealed increased levels in MG1655_Δ*pmrB* expressing PmrB_MG1655_ Leu_10_Pro compared with MG1655_Δ*pmrB* expressing PmrB_MG1655WT_ (Table 1).

Expression of PmrB_MG1655WT_ in LC711/14 was also able to reduce the colistin MIC of this strain from 4 to 1 mg/L, restoring colistin susceptibility and decreasing *pmrK* expression to a nearly basal level (Table 1).

Taken together, these results supported the hypothesis that the Leu_10_Pro amino acid substitution in PmrB is able to confer a Col-R phenotype in *E. coli* by upregulation of the *pmrHFIJKLM* LPS modification system.

### Investigation of the role of the amino acid at position 10 of PmrB

To further investigate the role of the amino acid residue at position 10 of PmrB of *E. coli*, additional mutants of PmrB_MG1655WT_ were generated, including PmrB_MG1655_ Leu_10_Gly and PmrB_MG1655_ Leu_10_Gln.

Complementation of MG1655_Δ*pmrB* with these mutants did not alter susceptibility to colistin, and the same mutants, expressed in LC711/14, did not modify the Col-R phenotype of this strain (Table 1). Expression experiments performed by qRT-PCR confirmed that the *pmrB* mutant derivatives (PmrB_MG1655_ Leu_10_Gly and PmrB_MG1655_ Leu_10_Gln) were expressed in *E. coli* MG1655_Δ*pmrB*, while *pmrB* expression was not detectable, as expected, in MG1655_Δ*pmrB* (data not shown).

Overall, these results suggested that the two mutated PmrB proteins were not functional, and that the residue at position 10 plays a crucial role in the structure and function of the PmrB sensor kinase.

### Prediction of the effect of PmrB mutations at position 10 at the protein level

**Figure 2:**
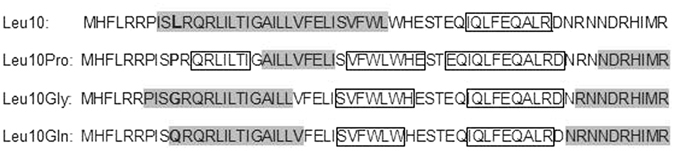
Secondary structure prediction of PmrB of MG1655 *E. coli* and its mutant derivatives. Analysis of the region encompassing the first 60 aa of the protein. In white are highlighted the coil regions. The helix regions are shaded in dark grey while the strand regions are boxed. The mutated aa in position 10 are boldfaced.

According to current knowledge on the PmrB protein structure^18^ position 10 is located into the amino-terminal protein portion including the cytoplasmic secretion signal (aa 1-13), which is a domain putatively involved in the delivery process of the protein into the cell membrane^19^ (Fig. 1). Comparison of the secondary structure of PmrB_MG1655WT_ with those of mutant derivatives analyzed in this work, using the YASPIN PREDICTION prediction tool, revealed that the mutations at position 10 could have a significant impact on the structure of the region encompassing the first 60 aa of the protein (Fig. 2), which includes the cytoplasmic secretion signal, the TM1 trans-membrane domain, and most of the periplasmic domain (Fig. 1).

The impact appeared to be higher with the Leu_10_Pro mutation, since the structure of the cytoplasmic domain and downstream regions were remarkably altered (Fig. 2), possibly due to the characteristic that proline is able to introduce kinks into alpha helices.

## Discussion

The emergence of colistin resistance in the clinical setting has become a matter of major concern given the primary role that colistin has regained in the treatment of infections caused by XDR Gram-negatives.

Knowledge concerning colistin resistance mechanisms among *E. coli* isolates of clinical origin remains limited. Recent studies, prompted by the discovery of *mcr*-type transferable colistin resistance determinants, have revealed that these determinants, which are highly prevalent among *E. coli* isolates from animals, can also be found among Col-R isolates from humans^10^. In animal isolates, mutations of the PmrAB proteins have also been associated with colistin resistance, although their role has not been formally demonstrated^13^.

This work provides for the first time a formal demonstration that a mutation in the PmrB sensor kinase can be responsible for colistin resistance in *E. coli*, and underlines the ability of *E. coli* clinical strains to evolve a Col-R phenotype through a number of different mechanisms. The strain was susceptible to other antibiotics and, in this case, colistin resistance did not pose any challenge to antimicrobial treatment. The Leu_10_Pro PmrB mutation has not been reported in other clinical strains, and its potential epidemiological impact in the clinical setting remains to be clarified. However, the strain belonged to a clonal lineage described to have pathogenic potential in humans and to be capable of clonal expansion^14^.

The Leu_10_Pro PmrB amino acid change is apparently responsible for constitutive activation of the PmrB sensor kinase, leading to downstream up-regulation of the *pmrHFIJKLM* operon encoding the major endogenous LPS modification system, which is under the control of the PmrAB signal transduction system^17^. The mechanism by which the Leu_10_Pro substitution alters the function of PmrB remains unknown. The mutation is close to the N-terminus of the protein, in the first cytoplasmic domain. Comparison of the predicted secondary structure of the amino-terminal moiety of wild-type PmrB with its mutant derivatives revealed a significant impact of mutations inserted at this position (Fig. 2), which could alter the conformation of downstream domains. These alterations could lead either to a constitutive activation of the sensor kinase, due to an unbalance of kinase and phosphatase functions (in the case of Leu_10_Pro), or to a loss of function (in the case of Leu_10_Gly and Leu_10_Gln).

Interestingly, mutations in the PmrB sensor kinase have previously been demonstrated to be involved in acquisition of a Col-R phenotype by activation of the endogenous LPS modification systems in other pathogenic species including *P. aeruginosa* ^18, 20^, *A. baumannii* ^21, 22^ and *K. pneumoniae* ^23, 24^, underscoring the role of PmrB as an important mutational target in evolution of colistin resistance in Gram-negative pathogens. Among the various PmrB mutations reported to be associated with this phenotype, a Leu_14_Pro mutation has been reported in *P. aeruginosa* ^18^. Since the Leu_14_Pro in *P. aeruginosa* and the Leu_10_Pro of *E. coli* are mutations located in the same cytoplasmic secretion signal domain of PmrB, this further supports the role that similar mutations may have in evolution toward colistin resistance.

## Methods

### Bacterial strains

The Col-R *E. coli* LC711/14 strain was isolated in 2014 from the urine of a patient admitted to the A. Manzoni Hospital in Lecco, Northern Italy. LC711/14 was previously reported as a Col-R *mcr-1*-negative clinical strain^15^. Identification of the strain at the species level was carried out using MALDI-TOF mass spectrometry (Vitek MS, bioMérieux, Marcy l’Etoile, France). The *E. coli* K12 strain MG1655 and its MG1655_Δ*pmrB* derivative (with the *pmrB* gene deleted) were from the Keio collection^25^. MLST analysis was carried out as previously described^26^, and ST was assigned in accordance with the *E. coli* MLST database (http://mlst.warwick.ac.uk/mlst/dbs/Ecoli/documents/primersColi_html).

### Antimicrobial susceptibility testing

Routine susceptibility testing of LC711/14 was performed using the Vitek 2 system (bioMérieux). MICs of colistin were determined by reference broth microdilution^27^. Results were interpreted according to the EUCAST breakpoints, version 7.1 (www.eucast.org). MICs of the complemented strains were carried out in microdilution broth adding 64 mg/L chloramphenicol to the culture.

### Recombinant DNA methodology, sequencing, site-directed mutagenesis and transcriptional analysis

Whole-cell DNA was extracted using the DNeasy Blood & Tissue kit (Qiagen GmbH, Hilden, Germany). The complete sequences of the *pmrAB* coding regions were amplified using primers and conditions reported in Supplementary Table S1. Primers were designed on the genome sequence of *E. coli* MG1655 (accession number: NC_000913.3). DNA sequences were determined for both strands at an external sequencing facility (GATC Biotech AG, Germany). The nucleotide and protein sequences were analyzed at the National Center for Biotechnology Information website (www.ncbi.nlm.nih.gov) using the Basic Local Alignment Search Tool (BLAST).

Plasmid pACYC-*pmrB*, used for complementation experiments and for site-directed mutagenesis experiments, is a pACYC184^28^ derivative carrying a cloned copy of the wild-type *pmrB* gene, amplified by PCR using primers pmrA-Ecoli_F and pmrB-ext-Ecoli_R (Table S1) from the genomic DNA of *E. coli* MG1655. The amplicon (1320 bp), covering the complete *pmrB* gene with its putative terminator and part of the upstream *pmrA* gene, was blunt-end cloned into pACYC184 digested with *Eco*RV. The authenticity of the cloned fragment was confirmed by sequencing. In the recombinant plasmid, the cloned *pmrB* gene was in opposite orientation of the tetracycline resistance cassette.

Site-directed mutagenesis of *pmrB* was carried out using the pACYC184-*pmrB* plasmid as a template. The primers (Table S1) were designed as described by Zheng *et al*.^29^. The authenticity of *pmrB* mutants was confirmed by sequencing on both DNA strands.

Recombinant plasmids were introduced into *E. coli* strains by electroporation, as previously described^30^. Transformants were selected on Mueller-Hinton agar (MHA) plates supplemented with 85 mg/L of chloramphenicol.

Expression of the cloned *pmrB* gene and mutant derivatives in *E. coli* MG1655_Δ*pmrB* transformed with the recombinant plasmids was verified by qRT-PCR using primers and conditions described in Table S1. Transcriptional analysis by qRT-PCR to measure expression of the *pmrK* gene was carried out as previously described^30^, using the pmrK_F and pmrK_R primers reported in Table S1. Expression of the *gapA* gene, evaluated with primers gapA_F and gapA_R (Table S1) was used as an internal standard. Normalization was performed against the *gapA* gene using the 2-ΔΔ*C*_*T*_ method (relative)^31^, and the values obtained were normalized against the value obtained with *E. coli* MG1655.

### Protein structure analysis

Prediction of the secondary structure of the PmrB protein was carried out with the YASPIN PREDICTION prediction tool, using non-redundant (NR) and DSSP-trainer databases (http://www.ibi.vu.nl/programs/yaspinwww/).

## Electronic supplementary material

Supplementary materials Table S1.
