# Supplementary material for: An allelic variant of the PmrB sensor kinase responsible for colistin resistance in an Escherichia coli strain of clinical origin
Source: Sci Rep. 2017 Jul 11;7:5071. doi: 10.1038/s41598-017-05167-6 (PMC5506025; doi:10.1038/s41598-017-05167-6)
Supplement: Supplementary file 1 — Supplementary materials Table S1. [file 41598_2017_5167_MOESM1_ESM.pdf]

Supplementary materials Table S1.

An allelic variant of the PmrB sensor kinase responsible for colistin resistance in an *Escherichia coli* strain of clinical origin

Antonio Cannatelli, Tommaso Giani, Noemi Aiezza, Vincenzo Di Pilato, Luigi Principe, Francesco Luzzaro, Cesira L. Galeotti, and Gian Maria Rossolini

**Application**	5’->3’	
**Cloning**	**Sequence**	**Cycling conditions (°C/s)***
pmrA-Ecoli_F	CGACATCTATAACTGGGACAATGAACC	D (95/30), A (58/30), E (72/120)
pmrB-ext-Ecoli_R	GAGGAGAGTGCAATGAAAAACCGTG	
**Mutagenesis**		
pmrB (t29c-Leu10Pro)_F	CAATATCGC<u>C</u>GCGCCAACGGCTGATATTGACC	D (95/45), A (62/45), E (72/240)
pmrB (t29c-Leu10Pro)_R	CCGTTGGCGC<u>G</u>GCGATATTGGTCGGCGCAGA	
pmrB (t29a-Leu10Gln)_F	CAATATCGC<u>A</u>GCGCCAACGGCTGATATTGACC	
pmrB (t29a-Leu10Gln)_R	CCGTTGGCGC<u>T</u>GCGATATTGGTCGGCGCAGA	
pmrB (c28g/t29cLeu10Gly)_F	CAATATCG<u>GGG</u>CGCCAACGGCTGATATTGACC	
pmrB (c28g/t29c -Leu10Gly)_R	CCGTTGGCG<u>CC</u>CCGATATTGGTCGGCGCAGA	
**Real-time PCR:**		
gapA-COLI_F	CGACAAATATGCTGGCCAGG	D (95/10), A (52/5), E (72/5)
gapA-COLI_ R	GTAGTAGCGTGAACGGTGGT	
pmrK-E coli_F	TGCGGAAATCAGTCGAGAAATGC	D (95/10), A (52/5), E (72/5)
pmrK-E coli_R	CGAAATAACGTAGCCCTAACAGATGG	
pmrB-E.coli-F	CATTTTCTGCGCCGACCAATATC	D (95/10), A (52/5), E (72/5)
pmrB-E coli_R	TCATGCCATAGCCAGAAGACGCTGATC	

Primer used in this work.*All conventional PCRs included an initial denaturation step of 180 s at 95°C, 30 cycles of denaturation, annealing, and extension at the reported temperatures and times, and a final extension step of 300 s at 72°C; all RT-qPCRs included an initial denaturation step of 300 s at 95°C and 40 cycles of denaturation, annealing, and extension at the reported temperatures and times. D, denaturation; A, annealing; E, extension.
